# Public Safety Personnel’s interpretations of potentially traumatic events

**DOI:** 10.1093/occmed/kqaa007

**Published:** 2020-02-10

**Authors:** R Ricciardelli, S Czarnuch, T O Afifi, T Taillieu, R N Carleton

**Affiliations:** 1 Department of Sociology, Memorial University of Newfoundland, St. John’s, Newfoundland A1C 5S7, Canada; 2 Department of Electrical and Computer Engineering and Discipline of Emergency Medicine, Memorial University of Newfoundland, St. John’s, Newfoundland A1C 5S7, Canada; 3 Department of Community Health Sciences, University of Manitoba, Winnipeg, Manitoba R3E 0W3, Canada; 4 Department of Psychology, University of Regina, Regina, Saskatchewan S4S 0A2, Canada

**Keywords:** Barriers to treatment, mental health, public safety personnel, traumatic events

## Abstract

**Background:**

Many public safety personnel (PSP) experience trauma directly or indirectly in their occupational role, yet there remain barriers to accessing care or seeking help.

**Aims:**

To understand how PSP interpret different potentially traumatic events and how perceived eligibility for being traumatized is determined among PSP.

**Methods:**

We analysed open-ended comments provided by over 800 PSP in a survey designed to assess the prevalence of post-traumatic stress injuries and other mental disorders.

**Results:**

We found evidence that a trauma hierarchy may exist among PSP. Certain experiences may be interpreted as more traumatic, based on both the event and the PSP role in the actual event. For example, involvement in a shooting may be interpreted as more traumatic than arriving on the scene later. Similarly, a single event may be deemed more traumatic than an accumulation of events. The role of the individual and social context in shaping experiences and interpretations of trauma may be largely ignored in line with confirmation biases.

**Conclusions:**

The role that individuals and social contexts play in shaping experiences and interpretations of trauma appear suppressed by perceptions of a trauma hierarchy, facilitating systematic discrediting or valuation of some experiences, therein evidencing that trauma is subjective and reinforcing barriers to care seeking. A trauma hierarchy may also propagate stigma and legitimize discrimination regarding mental health. We argue that recognizing, engaging with, and dismantling the perception of a trauma hierarchy may help create a respectful and open occupational culture supportive of mental health needs.

**Key learning points**

**What is already known about this subject:**

- Public safety personnel are exposed to trauma as part of their jobs.
- Yet, there remains a gap in knowledge of how public safety personnel experience and interpret potentially traumatic events.
- The current study is one of the first to explore how potentially traumatic event exposure shapes perception of trauma legitimacy.

**What this study adds:**

- We found evidence that a trauma hierarchy may exist among public safety personnel.
- Certain experiences may be interpreted as more traumatic, based on both the event and the public safety personnel role in the actual event.
- Similarly, a single event may be deemed more traumatic than an accumulation of events.

**What impact this may have on practice or policy:**

- Recognizing, engaging with, and dismantling the perception of a trauma hierarchy may help create a respectful and open occupational culture supportive of mental health needs.
- Further research is needed to understand the differential role of direct and indirect trauma.
- Such knowledge may reduce stigma concerning apparent interpretations of trauma.

## Introduction

Public safety personnel (PSP) are more likely to be exposed to trauma than the general population [1–5]. PSP work involves both direct (e.g. a PSP being attacked) and indirect (e.g. caring for a victim) exposures to potentially traumatic events (PTEs) [3]. We consider a PTE to be any incident involving the indirect or direct threat of sexual violence, death or serious harm [6]. Post-traumatic stress disorder (PTSD) is a well-recognized potential outcome from PTEs [6–8]; however, exposure to PTEs may also result in several other mental disorders [9–12]. The potential impact of multiple PTEs on mental health is evidenced in the Diagnostic and Statistical Manual of Mental Disorders (DSM-5) criteria for mental health disorder symptoms that recognize cumulative exposure to PTEs rather than just individual identifiable instances [6,8]. The DSM criteria may be important for people exposed to multiple PTEs, such as PSP who are working to ensure community safety.

Multinational evidence supports PSP having higher rates of mental disorders than the general public, which is believed to be associated with more frequent PTE exposures [13–18], and thus consistent with notions that exposure is a critical risk factor [6]. There is also evidence of significant barriers to accessing support or coming forward as potentially in need of treatment [19–21]. One key barrier may be a cultural perception within PSP that direct exposure to a PTE is a more recognized and accepted risk factor for mental disorders than indirect exposure [19,20]. Indirect exposure as a significant risk factor for problematic mental health is consistent with research and theory on vicarious trauma or secondary traumatic stress [22,23], which refer to negative impacts on mental health that result from exposure to the effects of a PTE experienced by another person [24–26]. There may be an implicit or explicit trauma hierarchy that influences how PSP frame their perceptions of legitimacy, stigma and treatment-seeking behaviour regarding PTEs and mental health.

In our study we unpack the idea of a trauma hierarchy, as described by PSP, and their interpretations of what constitutes trauma that would require seeking care. We provide evidence that an accumulation of direct or indirect exposure to PTEs can facilitate a trauma hierarchy that can negatively impact: (i) the prescribed legitimacy attributed to a singular event or a culmination of events; (ii) stigma; and (iii) treatment seeking. The current study is designed to complement that of Carleton and colleagues [3], who found that PSP, on average, reported being exposed to 11.08 (SD = 3.23) of 16 different forms of PTEs. PSP reported that the most commonly experienced events were sudden violent death (28%), accidental death (14%) and a serious transportation accident (14%). Our qualitative results contextualize Carleton and colleagues’ quantitative results using the descriptions provided by PSP. The resulting responses garner insight into PSP experiences with, and interpretations of, PTEs (see Table 2), giving voice to PSP who experience trauma. The results provide potentially critical avenues for understanding and engaging with PSP experiences, which we hope will ultimately help to reduce stigma and improve treatment seeking.

## Methods

An online survey was administered to PSP for 5 months between 2016 and 2017 [27]. The survey was approved by the University of Regina Institutional Research Ethics Board; #2016-107. Survey participants were given the Life Events Checklist for DSM-5 [28] which includes 16 close-ended answer options to choose in reporting traumatic events experienced and if they wanted to contextualize the events they could respond to the open-ended item ‘other, most traumatic event’. A total of 4441 participants completed the Life Events Checklist for DSM-5, but we focused specifically on the 284 PSP who provided open-ended responses. The sample included 110 female and 170 male participants (four did not provide a gender; see Table 1, for PSP occupational groups).

**Table 1.** Occupation of PSP

PSP occupational group	Total
Public Safety Communicators (e.g. call takers/911)	23
Other (coast guard, coroner, nursing, administrative, Union of Safety and Justice Employees, border services)^a^	8
Correctional services employees (Federal and provincial)	28
Paramedics	53
Firefighter (includes fire/paramedic, volunteer, search and rescue)	55
Municipal and Provincial police	59
RCMP	58

^a^The category of other was not analysed in Carleton *et al.* [21] (see Table 2).

**Table 2.** Prevalence of worst potentially traumatic exposures across Canadian public safety personnel categories

Type of worst exposure	Total, *n* (%)	Municipal/ provincial police, *n* (%)	RCMP, *n* (%)	Correctional workers, *n* (%)	Firefighters, *n* (%)	Paramedics, *n* (%)	Public safety communicators, *n* (%)
Life threatening natural disaster	77 (2)	10 (1)	15 (1)	7 (2)	17 (3)	19 (4)	9 (5)
Fire or explosion	123 (3)	22 (2)	26 (3)	– ^a^	51 (8)	16 (3)	6 (3)
Serious transportation accident	540 (14)	128 (13)	151 (14)	28 (6)	141 (22)	73 (14)	19 (10)
Serious accident at work, home or during recreational activity	130 (3)	25 (3)	26 (3)	22 (5)	29 (5)	20 (4)	8 (4)
Exposure to toxic substance	18 (1)	– ^a^	– ^a^	5 (1)	– ^a^	– ^a^	– ^a^
Physical assault	190 (5)	39 (4)	53 (5)	62 (13)	8 (1)	18 (3)	10 (5)
Assault with a weapon	245 (6)	84 (8)	92 (9)	40 (8)	8 (1)	14 (3)	7 (4)
Sexual assault	196 (5)	52 (5)	51 (5)	39 (8)	8 (1)	28 (5)	18 (10)
Other unwanted or uncomfortable sexual experience	53 (1)	9 (1)	9 (1)	16 (3)	– ^a^	14 (3)	– ^a^
Combat	43 (1)	16 (2)	10 (1)	8 (2)	6 (1)	– ^a^	– ^a^
Captivity	25 (1)	7 (1)	7 (1)	9 (2)	– ^a^	– ^a^	– ^a^
Life threatening illness or injury	255 (7)	60 (6)	58 (6)	57 (12)	30 (5)	33 (6)	17 (9)
Severe human suffering	272 (7)	69 (7)	45 (4)	33 (7)	55 (9)	62 (12)	8 (4)
Sudden violent death	1086 (28)	324 (33)	344 (33)	114 (24)	138 (22)	115 (21)	51 (28)
Sudden accidental death	542 (14)	114 (12)	129 (12)	30 (6)	130 (20)	110 (21)	29 (16)
Serious injury, harm or death you caused to someone else	81 (2)	32 (3)	28 (3)	4 (1)	6 (1)	10 (2)	– ^a^

Table is from Carleton et al. [21].

–^a^Not presented because of insufficient sample size (i.e. *n* < 5).

PSP responses were thematically analysed after the data set was imported into NVivo (see [19,29] for detailed information about analysis). Analyses used a semi-grounded constructed approach with axial coding, which allowed coders to disaggregate and reclassify emergent themes until a cohesive categorization of the topics discussed by PSP in the comments emerged. We edited PSP responses when necessary for grammar and spelling.

## Results

Participants who responded ‘other’ often felt a need to validate or justify why they found their most traumatic event to be so impactful or to describe the situation around the event. Our participants suggested that not all PTEs are considered equally impactful; instead, some PTEs appear to be deemed more traumatic than others. Participants also reported expecting that suffering as a result of a traumatic experience would be proportional to the extent the experience is interpreted as traumatic, regardless of how the individual was affected by the event. Suffering based on an experience that is perceived as widely accepted as traumatic among PSP was deemed legitimate, whereas the same amount of suffering resulting from an experience perceived as unlikely to be traumatic for most PSP (e.g. a routine occurrence) was designated illegitimate. This central finding implies a hierarchy of trauma exists within the PSP collective consciousness; a hierarchy independent of the actual incident of trauma experienced (see Table 2). Instead, the hierarchy appears to be constructed based on *how* the trauma is experienced. Ranking our PSP responses from most to least traumatic suggests the most traumatic PTEs are:

(i) Being first on the scene (e.g. an accident, violence or any incident)
(ii) Responding to the scene, but not as the first on scene
(iii) Managing the scene
(iv) Accumulated exposure to traumatic events over time, but without an ‘anchor event’
(v) Indirect exposure to PTE
(vi) Unsupported and ‘old’ trauma (e.g. trauma that is not recognized by PSP colleagues)

The hierarchy reflects notions that certain events are considered more traumatic. Moreover, PSP reported that specific incidents, those involving the handling of dead bodies; the death of a child or by suicide; requiring the provision of direct care (e.g. cardiopulmonary resuscitation); and motor vehicle collisions, were particularly dire to manage. Our participant responses indicate a hierarchical trauma wherein PSP who are exposed to trauma more directly are collectively identified as the ‘most legitimate’ trauma; as such, garnering the most understanding and seeking support or treatment appears warranted. Conversely, exposure to events in ways considered less directly traumatic, as suggested by our respondents, can be interpreted as not legitimate (or their suffering and thus need for treatment is unwarranted). PSP whose exposure is increasingly less direct to the trauma may be less likely to acknowledge their need for support or treatment, regardless of the actual impact these events may have.

The first three items in the trauma hierarchy appear intuitive; nevertheless, participants expressed significant difficulty in describing trauma that was cumulative (ranked fourth). For example, Royal Canadian Mounted Police (RCMP) officer participants describe struggling to acknowledge their work-related mental illnesses because their suffering did not stem from one specific event:

I believe a series of traumatic events over a longer period have contributed to my illness(es) rather than one specific incident… It is harder for us to acknowledge mental illness when there is no anchor event (2520, RCMP, did not disclose gender).

…over the years, the repeated trauma exposure from being a cop eventually proved too much for me to bottle up any more… Because I got PTSD as a result of regular police work (though I did experience a lot of serious incidents, more than most I think) I felt like I had failed as a cop, and that I was less capable than I thought… (984, RCMP, male).

These officers’ words support that only under certain circumstances, where an individual has experienced what is understood to be legitimate trauma, is an individual’s resultant suffering considered to be warranted and deserving of intervention. Officer 984’s statement that he felt like a failure as a cop because his PTSD was ‘a result of regular police work’ demonstrates the belief that developing PTSD should have never happened without a significant exposure to a specific incident, which facilitates the notion that he was perceived less capable. The extremely frequent direct and indirect exposures to PTE for PSP fit with contemporary notions that cumulative exposure can cause significant symptoms; however, that does not require that all exposures are perceived as comparable and by extension none were significant. Instead, there may be so many significant exposures that how symptoms are understood and presented becomes more complex.

Participants reported that PSP who experience PTEs require mental health support even if they have not responded to what, they feel, would be understood as a legitimately traumatic event. An officer explains:

Supervisors should be EXTREMELY cognizant of their employees’ calls to make sure they receive proper and thorough mental health support, not just for shootings, multiple casualties, gruesome deaths, child related crimes or deaths. Front line workers would benefit from education re Cumulative PTSD, so they know when and how to get help (3777, police, female).

Her response indicates the potential importance of understanding the effects of accumulated stressful events; similarly, a communications official reported: ‘…it would be nice if there was designated time, if needed, for us to go make a quick call for support to someone who truly understands the job’ (4450, communications, female). The participants provide evidence that PSP may be offered support for only some experiences, whereas experiences that produce negative feelings and are not considered to be recognized and accepted sources of trauma appear disregarded.

Indirect exposure to PTEs, fifth on the hierarchy, provides a different approach to understanding traumatic exposure. Many PSP are exposed to direct and indirect (i.e. vicarious) trauma; as such, many reported feeling their experiences are discounted or illegitimate unless the experience is direct and timely. For example, a male parole officer reported feeling his vicarious trauma was illegitimate because he is not considered to be a first responder and not directly exposed to most events (3904, correctional services, male). He explains that working extensively with parolees, and their families and victims can also be taxing for parole officers (3904, correctional services, male). A probation officer corroborated: ‘Being in a profession where you read violent reports and supervise violent individuals is very traumatic’ (4239, correctional services, male). A 911 police dispatcher explained:

Working in a communication centre that covers a large area I am exposed daily to constant negative situations. Nobody calls 911 asking to speak to the police because they are having a good day. For me it’s the constant inflow of negative situations that all add up to become the problem, not one specific incident… (4722, communications official, male).

He explains that not all occupational stress injuries stem from a singularly experienced anchor event, but may result after an accumulation of indirect exposures. Together, respondents 3904 and 4722 suggest that a focus on what respondents have seen or directly experienced excludes the experiences of PSP who do not directly witness events including being the first on scene. A communications official, in this context, states ‘…here in the communication centre I rarely see anything, but I hear everything’ (4722, communications, male). Another communications official indicated that her group is overlooked during incident debriefings and when communicators report suffering from an occupational stress injury, the injury is often not taken seriously:

When we take horrible calls we are often overlooked in a debriefing held afterwards. We constantly deal with screams in our ears, crying, sounds of horror, fear, death, suicides, assaults, robberies, murders, info being thrown our way, we handle such chaos with professionalism and empathy. If we make 1 mistake it could cause someone their life... Most employees in my job have PTSD but get ridiculed for being labelled attention seeker or making it all up. As you can tell we are frustrated (9123, communications, female).

She reports significant dissatisfaction with the extent to which her work is understood as potentially traumatic; indeed, communicators may feel greatly affected by their work and nonetheless feel their potential mental health risks are minimized by other PSP. Correctional staff also endure indirect exposure to PTE; they hear the details and descriptors of various offenses, but needing to remain empathetic as they help teach those in their custody ways to refrain from re-offending. PSP suffer from vicarious trauma due to the nature of their work, yet feel this vicarious trauma is not considered to be a legitimate source of suffering.

Regarding the sixth point on the hierarchy, unsupported and ‘old’ trauma, respondents also indicated feeling ill-effects of events that contextualize the PTE rather than the critical incident. Participants may not believe their feelings are legitimate in the absence of a direct relationship between symptoms and a specific PTE. A male Emergency Medical Technician (EMT) explained feeling less affected by the nature of the calls he attends and more affected by the lack of support he receives in recuperating from those calls: ‘Workload is a huge issue. People need time to decompress from calls and downtime to eat, drink and use the washroom’ (1799, field medicine, male). The EMT’s words suggest that some people may be able to effectively deal with PTE if given appropriate support (e.g. time between calls). Unfortunately, in many PSP occupations, time may not be available due to the inherent nature of the work: ‘[correctional] officers involved in events are usually back to work [on] the next shift with little or no decompression time’ (1555, correctional services, male). Similarly, a work-related event considered to be non-traumatic, and unrelated to PTEs can also serve to negatively affect a PSP, a parole officer noted:

The cumulative effect of dealing with difficult people over the years, the organization that I work for and the ongoing effect of hearing about horrible things happening to people seems to have built up over time but I was dealing with it all well (I think) until recently an event at work (job change that was NOT wanted or requested) has caused me to feel unvalued and unappreciated (3719, Correctional Services, female).

PSP appear aware that suffering due to a PTE need not look a specific way and may not immediately follow a single specific event. PSP clearly reveal that an event many not affect a person negatively until years later, and at this point, the individual may not feel legitimized in seeking help. A police officer, echoing others, noted, ‘There are cumulative effects of first responders’ work that do not affect you at the time they occur, however seem to come back to mind several years later’ (4412, police, female). Similarly, an RCMP officer contributed that, despite many years of attempting to deal with traumatic events through compartmentalization, unexpected feelings can surface, often due to an unrelated and non-traumatic event. A female firefighter described how the temporal proximity of the event affects the perceived legitimacy of the suffering it induces, echoing other respondents in indicating that that there may be an expiry date on how long and when an individual is allowed to feel traumatized, suggesting that feeling affected by events that transpired in the past may not be looked upon as acceptable.

## Discussion

Extending the work of Carleton *et al.* [3], we document what appears to be evidence of a trauma hierarchy from responses provided by PSP; specifically, suffering is considered most legitimate if the exposure to trauma is direct, rather than indirect or cumulative. Suffering in response to events that PSP perceive as traumatic, but are not thought to warrant suffering, may cause PSP to deny or avoid their feelings, and may deter treatment seeking. PSP expressed a belief that the suffering associated with an event should only take place immediately after an incident. Unfortunately, the trauma exposure hierarchy, in conjunction with the constraints placed when suffering must occur to be legitimate, may be real potential barriers to treatment seeking and problematic reinforcers of mental health stigma.

Our study is the first to explore how direct and indirect PTE exposures shape perceptions of perceived traumatic experience legitimacy through unstructured, open-ended survey items. Through an anonymous survey, our respondents expressed their opinions about their perceptions of exposure to trauma and PTEs without fear of sanctions or stigma and potential impact on career advancement, though we underscore recruitment and self-selection biases as design limitations that should be considered when interpreting our results. The use of an online survey platform is also inherently restrictive in that participants may have had other relevant contributions that were left unexpressed due to a lack of prompting or perceived opportunity.

The hierarchy of trauma exposure corresponds to evolving understandings of mental health and mental disorders. For example, discussions of differentiating events as sufficient to cause PTSD were associated with the DSM-III [6] and involved potentially problematically subjective assessments (i.e. events ‘outside the range of usual human experience’, p. 236, and would ‘evoke significant symptoms of distress in almost everyone’, p. 238). DSM-5 does not include the same subjective assessment challenges as DSM-III and instead focuses on exposures involving ‘actual or threatened death, serious injury, or sexual violence’ (p. 271). Our results support the current DSM-5 [6] framing and, in particular, support the DSM-5 inclusion of indirect exposure to PTE as potentially problematic [6]. Our results also support the need to engage in additional research to understand the differential role of direct and indirect exposure to trauma, not only on the individual as a result of the exposure [22], but on how perceptions of differences may impact stigma and treatment seeking. That understanding may help to inform interventions designed to reduce stigma among PSP and increase treatment seeking. Understanding PSP perceptions of a trauma hierarchy that delineates levels of legitimacy for psychological reactions should help PSP organizations to design more effective stigma reduction interventions, promote help-seeking behaviours and inform perpetual improvements for treatment protocols. By recognizing that PTEs that are considered illegitimate can still result in the development of mental disorders if not treated, early intervention can be encouraged and rates of untreated mental illness may be reduced, promoting a healthier workforce and reduce lost workdays.

## Funding

R.N.C.’s research is supported by the Canadian Institute of Health Research (CIHR) through a New Investigator Award (FRN: 285489). The current research is also supported by a CIHR Catalyst Grant (FRN: 162545). T.O.A.’s research is supported by a CIHR New Investigator Award and Foundation Scheme Award. This research was funded in part by the Ministry of Public Safety and Emergency Preparedness through the Policy Development Contribution Program.
